# WEATHER AND AGING RESILIENT MODEL (WARM) FOR AFFORDABLE HOUSING

**DOI:** 10.1093/geroni/igae098.1609

**Published:** 2024-12-31

**Authors:** Holly Dabelko-Schoeny, Smitha Rao, Sean Bartlett

**Affiliations:** The Ohio State University, Columbus, Ohio, United States; The Ohio State University, Columbus, Ohio, United States; Central Ohio Area Agency On Aging, Columbus, Ohio, United States

## Abstract

Low-income housing residents, especially older adults with disabilities, face heightened risks during weather-related disasters. This study examines how older adults in affordable housing experience extreme weather, and roles and experiences of service coordinators in preparedness and response. Utilizing a mixed methods community-based participatory case-study design, we conducted a cross-sectional survey (N=40) and focus group (N=26) with residents in one senior affordable housing property in Central Ohio. Another survey (N = 8) and focus group (N = 11) included service coordinators and supervisors working within eight affordable housing properties in the region. Among residents, a third reported fair or poor health and over half (53%) did not drive. Most (73%) had experienced extreme weather events, with snow/ice storms and hurricanes being most common and extreme heat the most recent. Nearly 70% experienced power outages, the average outage lasted a week, and 40% did not have an evacuation plan. Service coordinators experienced extreme heat, fire, and severe snow/ice and few had advance-warning to prepare residents for extreme weather. Thirty-eight percent lost power in their buildings, and only half had reliable information and communications during the event. Seventy-five percent were somewhat or very concerned about disasters and the same number reported their residents were unprepared. Seventy-five percent lacked emergency preparedness training and 63% were not confident in their ability to evacuate residents. Focus groups among residents and coordinators revealed challenges in communication, transportation, sheltering, power sources, and evacuation. Findings are critical to the co-development of a disaster preparedness toolkit for affordable housing communities.

